# Supplementary material for: Comparative NMR Metabolomics Profiling between Mexican Ancestral & Artisanal Mezcals and Industrialized Wines to Discriminate Geographical Origins, Agave Species or Grape Varieties and Manufacturing Processes as a Function of Their Quality Attributes
Source: Foods. 2021 Jan 13;10(1):157. doi: 10.3390/foods10010157 (PMC7828614; doi:10.3390/foods10010157)
Supplement: Supplementary file 1 [file foods-10-00157-s001.pdf]

Supplementary Materials

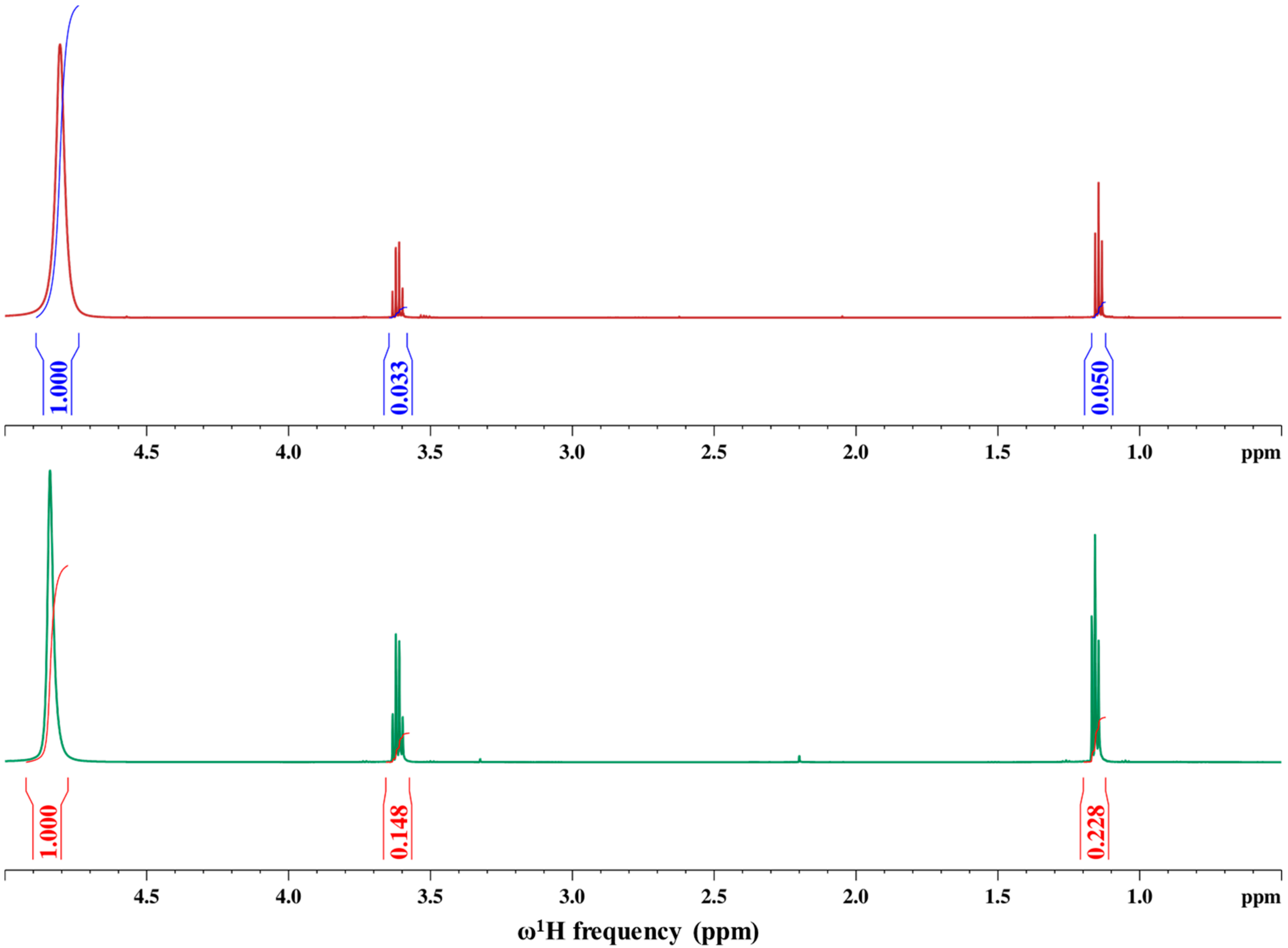

Figure S1. Standard direct-excitation one-dimensional proton nuclear magnetic resonance spectra of a wine (red) and mezcal (green) representative sample. Signal integration of intense water (4.83 ppm) and ethanol CH_2 (3.51 ppm) - CH_3 (1.14) spin systems is the starting point to predict effective power level of suppression off-resonance shaped pulses, applied in the {^{1}Hwater_presat NMR}: 1D single pulse NOESY experiments, needed to produce NMR outliers (Figure 1, Main text) used

in MSA. Relative ethanol signal integration with respect water resonances reflect the different %ABV content in both spirits, that in turn defines the off-resonance shaped pulses amplitude differences in each case: 8.19 x10^{-4} W (for wine samples) and 1.18 x10^{-3} W (for mezcal samples).

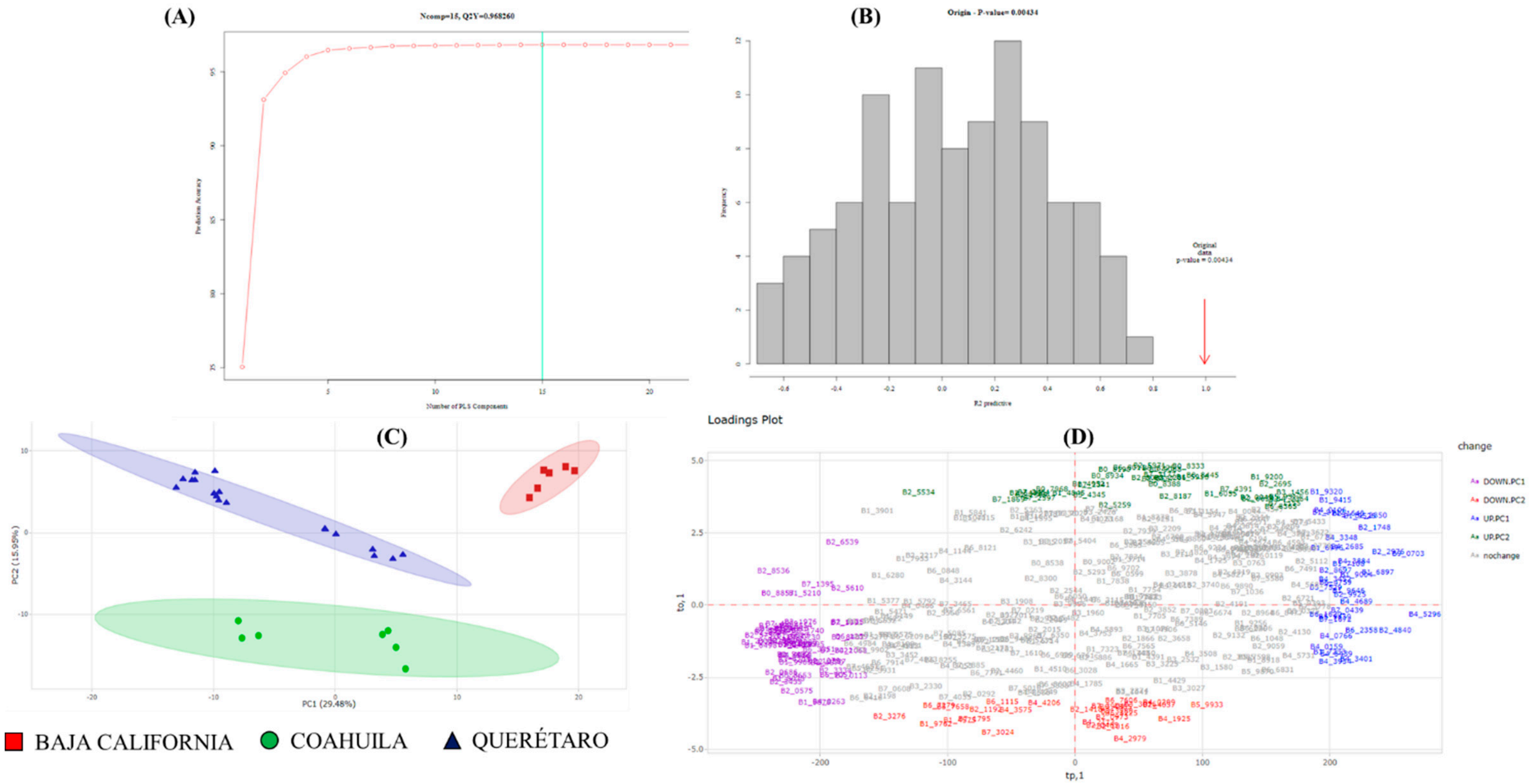

Figure S2. Prediction accuracy curves as a function of PLS components, permutation tests, Principal Component Analysis (PCA) obtained from the same NMR data matrix and OPLS-DA loading plots of wines' regional supervised discriminative analysis presented in Figure 2 top, main text.

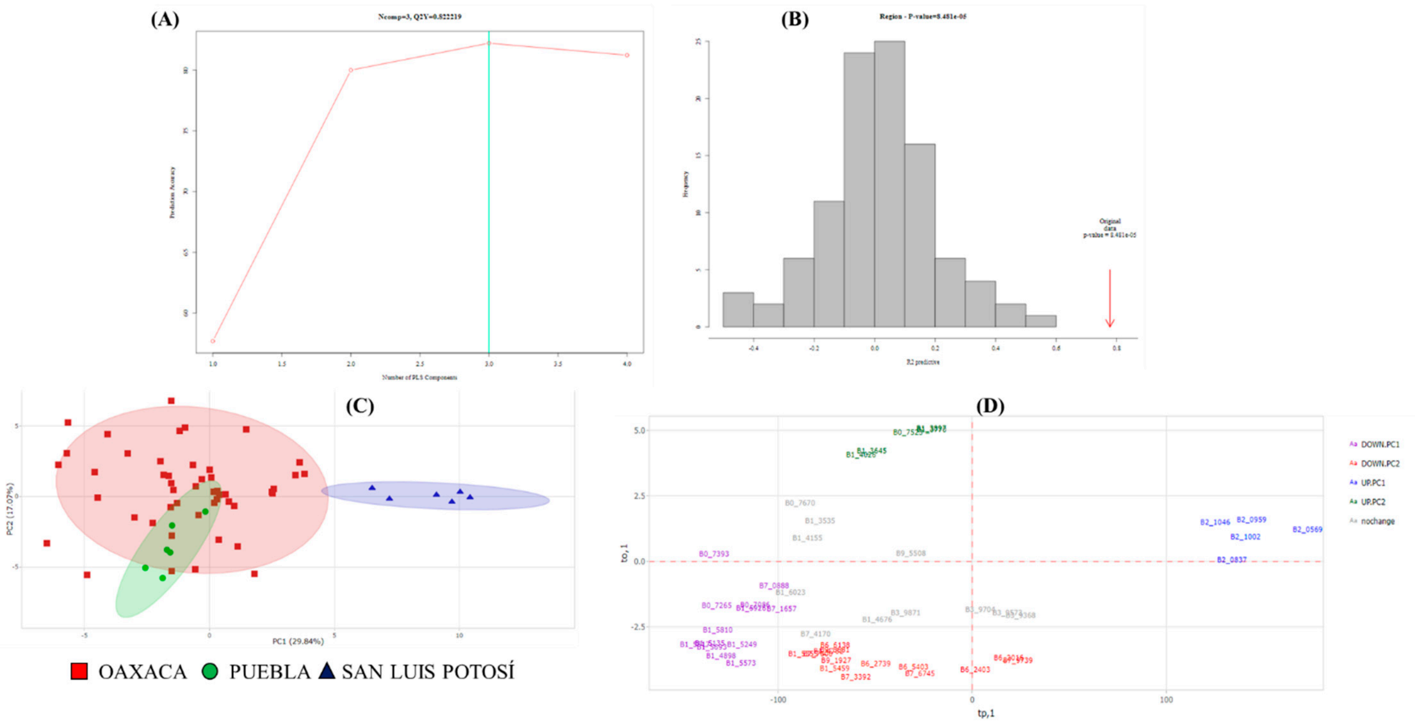

Figure S3. Prediction accuracy curves as a function of PLS components, permutation tests, Principal Component Analysis (PCA) obtained from the same NMR data matrix and OPLS-DA loading plots of mezcals' regional supervised discriminative analysis presented in Figure 2 bottom, main text.

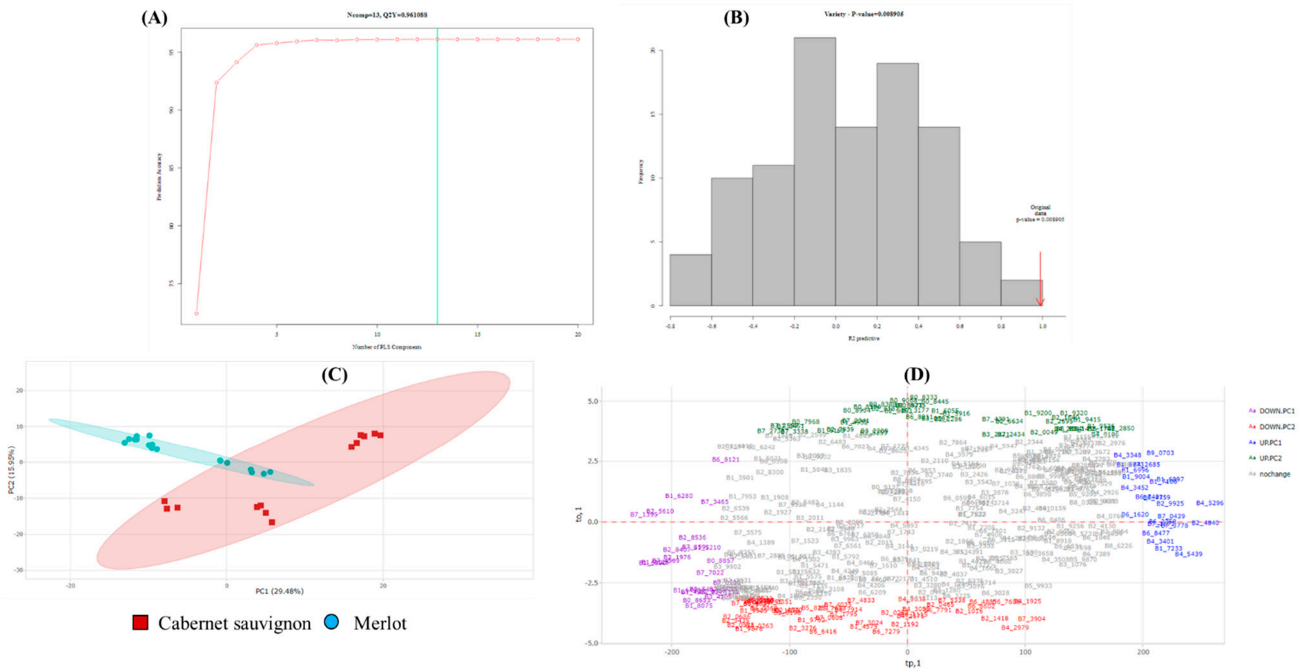

Figure S4. Prediction accuracy curves as a function of PLS components, permutation tests, Principal Component Analysis (PCA) obtained from the same NMR data matrix and OPLS-DA loading plots of wines' varieties supervised discriminative analysis presented in Figure 3 top, main text.

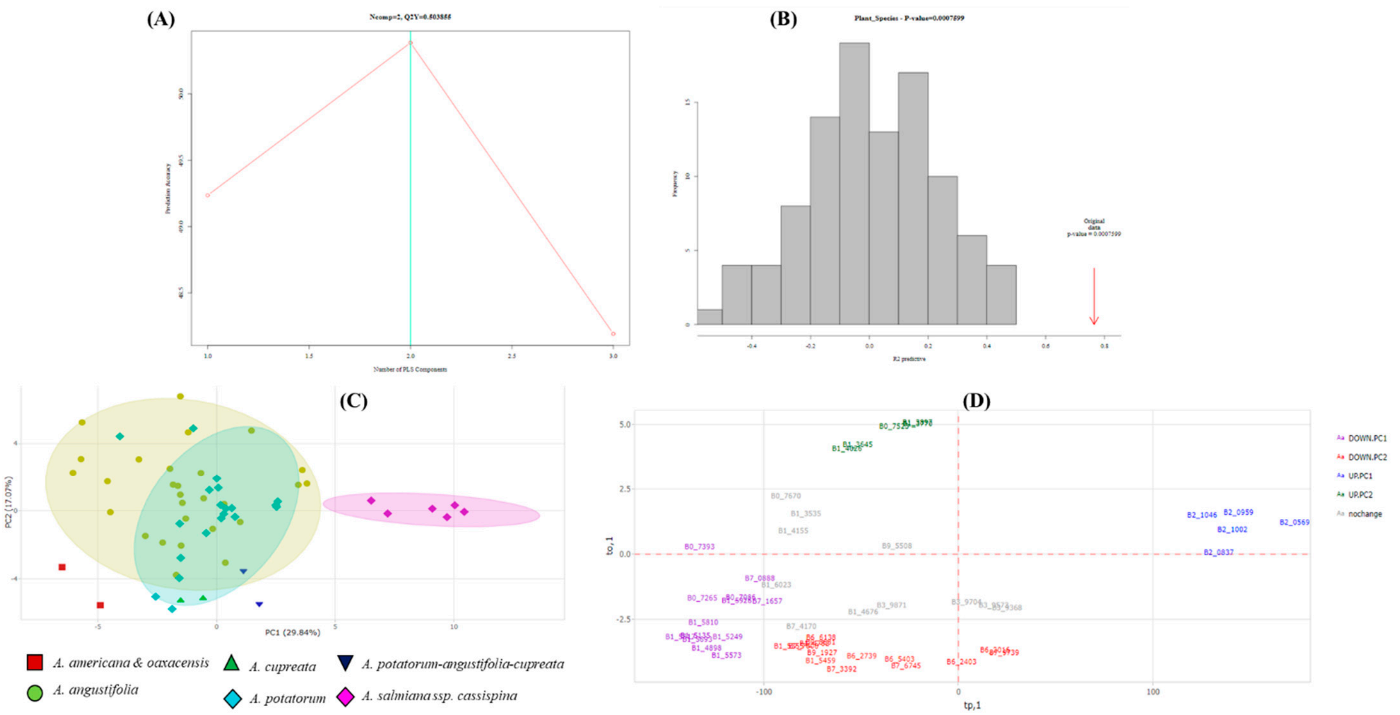

Figure S5. Prediction accuracy curves as a function of PLS components, permutation tests, Principal Component Analysis (PCA) obtained from the same NMR data matrix and OPLS-DA loading plots of mezcals' species supervised discriminative analysis presented in Figure 3 bottom, main text.

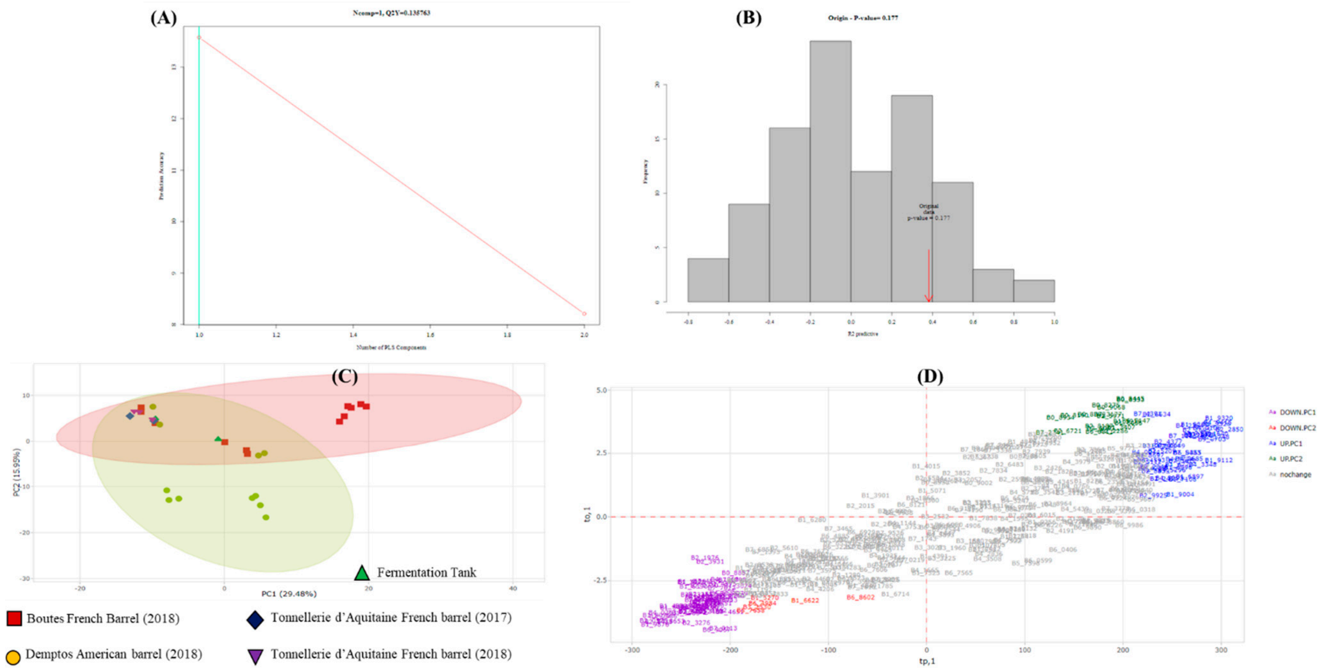

Figure S6. Prediction accuracy curves as a function of PLS components, permutation tests, Principal Component Analysis (PCA) obtained from the same NMR data matrix and OPLS-DA loading plots of wines' ageing processes supervised discriminative analysis presented in Figure 4 top, main text.

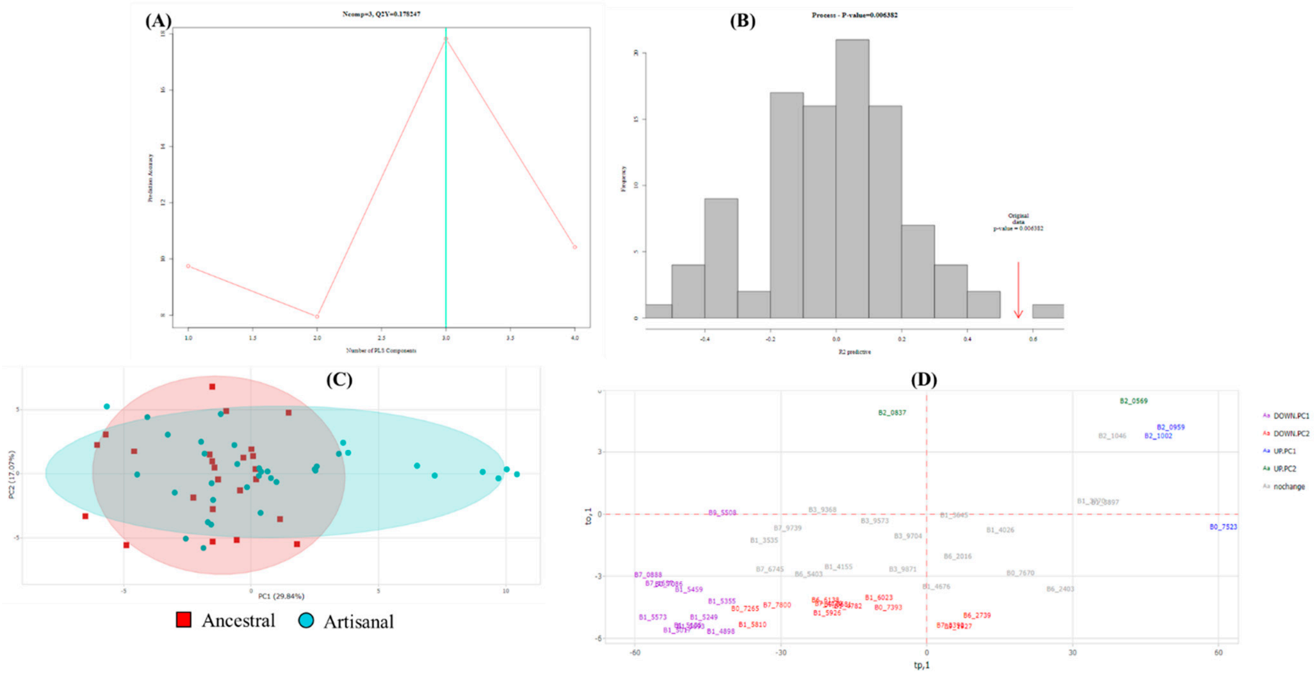

Figure S7. Prediction accuracy curves as a function of PLS components, permutation tests, Principal Component Analysis (PCA) obtained from the same NMR data matrix and OPLS-DA loading plots of mezcals' manufacturing processes supervised discriminative analysis presented in Figure 4 bottom, main text.
